# Pharmacokinetic study on pradofloxacin in the dog – Comparison of serum analysis, ultrafiltration and tissue sampling after oral administration

**DOI:** 10.1186/1746-6148-9-32

**Published:** 2013-02-14

**Authors:** Gregor Hauschild, Karl Rohn, Eva Engelhardt, Martin Sager, Jendrik Hardes, Georg Gosheger

**Affiliations:** 1 Department of Orthopedics and Tumororthopedics, University Hospital of Münster, Albert-Schweitzer-Straße 33, Münster, 48149, Germany; 2 Department of Biometry, Epidemiology and Information Processing; University of Veterinary Medicine Hannover, Hannover, Germany; 3 Central Animal Laboratory, Heinrich Heine University of Düsseldorf, Düsseldorf, Germany; 4 LESIA Center for Veterinary Medicine, Hannov, Germany

## Abstract

**Background:**

Pradofloxacin, a newly developed 8-cyano-fluoroquinolone, show enhanced activity against Gram-positive organisms and anaerobes to treat canine and feline bacterial infections. The purpose of this cross-over study was to measure the unbound drug concentration of pradofloxacin in the interstitial fluid (ISF) using ultrafiltration and to compare the kinetics of pradofloxacin in serum, ISF and tissue using enrofloxacin as reference.

**Results:**

After oral administration of enrofloxacin (5 mg/kg) and pradofloxacin (3 mg/kg and 6 mg/kg, respectively), serum collection and ultrafiltration in regular intervals over a period of 24 h were performed, followed by tissue sampling at the end of the third dosing protocol (pradofloxacin 6 mg/kg). Peak concentrations of pradofloxacin (3 mg/kg) were 1.55±0.31 μg/ml in the ISF and 1.85±0.23 μg/ml in serum and for pradofloxacin (6 mg/kg) 2.71±0.81 μg/kg in the ISF and 2.77±0.64 μg/kg in serum; both without a statistical difference between ISF and serum. Comparison between all sampling approaches showed no consistent pattern of statistical differences.

**Conclusions:**

Despite some technical shortcomings the ultrafiltration approach appears to be the most sensitive sampling technique to estimate pharmacokinetic values of pradofloxacin at the infection site. Pharmacokinetics – Pradofloxacin – Ultrafiltration – Dog – Oral Administration.

## Background

Since first introduced in the 1960s, fluoroquinolones have undergone continual modification [1]. The third generation of this substance group is not only characterised by its broad-spectrum activity against gram-negative species but also by an enhanced activity against gram-positive bacteria and a high efficacy against anaerobes. In addition its prolonged serum half-life permits a convenient once daily dosage [1-4]. Pradofloxacin, an 8-cyano-fluoroquinolone and third generation drug, is has been developed exclusively for the veterinary market. Compared to the well-established enrofloxacin, which was the first fluoroquinolone exclusively developed for the veterinary market, its structure differs in a bicyclic basic ammonium derivative in position C-7 as well as a cyano-group in position C-8. The bicyclic amine is mainly responsible for the increased potency of the substance, while the cyano-group is responsible for its enhanced activity against first- and second-step fluoroquinolone resistant bacteria [1].

Since the majority of bacterial infections are extracellular, optimisation of the antimicrobial drug concentration at the site of infection, i.e. in the interstitial fluid (ISF), is important to reach a therapeutic effect [5]. Thus, investigation of the concentration of unbound antimicrobial in the ISF is of great meaning and important to predict therapeutic efficacy. Ultrafiltration enables minimally invasive measurement *in vivo* of these unbound substance concentrations in the ISF. In essence consisting of a biocompatible hollow micro-fiber with a semipermeable membrane and a sample vial under vacuum, this system enables continuous, selective uptake of the substance from the examined tissue by using negative pressure and provides continuous tissue sampling in awake, unrestrained animals. The system produces a filtrate, whose molecular components do not exceed a size of 30,000 d. Thus, larger proteins and cellular components are filtered out, and direct analysis of unbound and thus effective drug is possible. Disadvantages of the microdialysis technique such as small sample volumes and low concentrations collected as well as the system immanent continuous perfusion of the probe with fluid which does not allow equilibrium between ISF and perfusion fluid [6,7] are not supposed to appear using the ultrafiltration technique. It was the aim of the present study to investigate the pharmacokinetics of unbound pradofloxacin using ultrafiltration and to compare the outcome of this sampling technique to the results gained by the analysis of standard serum and tissue samples.

## Methods

Animal experiments were conducted under an ethic committee approved protocol in accordance with German federal animal welfare legislation (Az 50.05-230-84/06), which is in compliance with the guidelines outlined in the NRC Guide for the Care and Use of Laboratory Animals. All animals were housed in groups of two animals at the Central Animal Laboratory of the Heinrich-Heine-University of Duesseldorf, Germany, University Hospital, and all procedures were performed in that same facility.

### Study design

The study followed a three-period three-treatment cross-over design. Six healthy female beagle dogs (B. Bomholt, 44579 Castrop-Rauxel, Germany) with a body weight ranging from 11.5 to 16 kg were included. Treatment started with administration of enrofloxacin 5 mg/kg, followed by pradofloxacin 3 mg/kg and pradofloxacin 6 mg/kg. Each substance was given orally q24h for 6 days followed by a washout period of at least seven days between each treatment. Serum and ISF sampling started on the fifth day of treatment at 0 (pretreatment) 0.5, 1, 2, 4, 8, 12 and 24 hours after administration. When necessary, the dose was adapted to the bodyweight of the test animals by breaking the tablet into two or four equal parts. By the end of the study, each dog had received each of three treatments containing enrofloxacin 5 mg/kg, pradofloxacin 3 mg/kg and pradofloxacin 6 mg/kg.

### Blood collection

Blood was collected from the Vena cephalica antebrachii using a peripheral intravenous cannula (Braun, Melsungen, Germany) and commercially available plastic tubes containing a clot activator (Monovette, Sarstedt, Nümbrecht, Germany). This was followed by centrifugation at 2.500 g for 10 min. Serum was separated and frozen at -18°C until analysis.

### Tissue sampling

Sacrification of the experimental animals was performed within 1 - 1.5 h after the last dose of drug was administered. This was followed by tissue sampling from bone, cartilage, skin, muscle, fat, liver, kidney and cerebrospinal fluid (CSF). The bone and cartilage samples were taken from the stifle joint (trochlea ossis femoris); skin, muscle and fat were collected from the abdominal wall (Musculus rectus abdominis, intra-abdominal fat). Wedge biopsies were performed to collect liver and kidney samples, whereas CSF was taken by puncture of the subarachnoid space. All tissues were collected as double samples, saved native in Falcon tubes (polypropylene conical tubes 50 ml, Becton Dickinson labware, USA) and frozen immediately at -18°C. CSF was saved in Eppendorf tubes (Eppendorf microcentrifuge tubes, 2.0 ml, Eppendorf, Germany) and handled like the tissue samples.

### Ultrafiltration (collection of ISF)

Subcutaneous sampling of ISF was performed parallel to blood collection using an in vivo ultrafiltration system (BASi Inc., West Lafayette, IN, USA) in accordance to the procedure published by Bidgood and Papich (2005)[8]. This system consists of three main components: the ultrafiltration (UF) probe, a hub assembly and a vacutainer. The UF-3-12-probe had three loops of membrane, and each membrane loop contained 12 cm of semipermeable membrane which offered 36 cm of available membrane surface for ultrafiltration. The semipermeable membrane of this probe was characterised by a molecular weight cut-off value of 30 kd, providing the collection of samples of unbound drug fraction. In modification of the established procedure the non-permeable part of the probe which extended to the exterior of the animal was fixed with tape-flaps sutured to the skin and was additionally secured by a feeding tube which covered the probe to prevent bending. After connecting the probe with the vacutainer, the system was protected with a jacket made to fit for each dog.

### HPLC analysis of serum, ISF and CSF

We investigated the test substance pradofloxacin (Veraflox®, Bayer Animal Health GmbH, Germany) with a molecular weight of 396.4 Da as well as the reference substance enrofloxacin (Baytril®, Bayer Vital GmbH, Germany) with a molecular weight of 359.4 d with a limit of quantification (LOQ) of 0.025 μg/ml using an identical approach. For HPLC analysis a Turbulent Flow Chromatography system 2300 HTLCTM (Cohesive Technologies Inc.) with auto injector CTC HTS PAL (CTC Analytics AG) coupled to a tandem mass spectrometer Sciex API 365 (Applied Biosystems) was used. As extraction column we used a HTLCTM Cyclone 1 × 50 mm, 60 μm polymer (Thermo Fisher). Serum and ISF samples were centrifuged at 4°C and 15,000 g for 10 min, and an aliquot was transferred into an autosampler vial. After addition of the internal standard (pradofloxacin-d4 or enrofloxacin-ethyl-d5 and ciprofloxacin-piperazyl-d8), 20 μl were injected into the HPLC. Sample pre-treatment was performed directly in the HPLC system. The undiluted sample was injected and transferred by the isocratic pump with a high flow rate (5 ml/min of an acidic solution of ammonium acetate containing 0.77 g ammonium acetate and 1.5 ml trifluoroacetic acid in 1 l water) to the extraction column. After switching to the elution position the analyte was eluted with the mobile phase (gradient from 90% acidic solution of ammonium acetate and 10% acetonitrile to 100% acetonitrile in 5 seconds, flow rate 1.5 ml /min) and transferred to the mass spectrometric detector (split approximately 1:10). The analyte was determined in the multiple-reaction-monitoring mode. The calculation of the concentration was performed by comparison with matrix-matched standards containing the internal standard [9].

### HPLC analysis of tissue samples

Tissue samples of muscle, fat, liver and kidney (1 g) were extracted by homogenisation with 10 ml of a mixture of acetonitrile (500 ml/l) and 0.1 ml/l formic acid. After centrifugation of the suspension for approximately 10 min, an aliquot of the liquid phase was passed through a 0.2 μm filter. One ml of the filtrate was transferred into an auto-sampler vial, and internal standard solution (containing pradofloxacin-d4) was added. Analysis was performed by Turbulent Flow Chromatography / Tandem Mass Spectrometry as described above, but in addition, after switching to the elution position, the analyte was eluted to an analytical column (Chromolith Speed Rod, 50 × 4.6 mm RP 18e, Merck) and then transferred to the mass spectrometer [10].

Samples of skin (100 to 200 mg) and cartilage (10 – 60 mg) were digested by shaking with 0.5 ml Proteinase K solution for 48 h at 55°C. The resulting suspension was acidified with 1.5 ml diluted formic acid and treated in an ultrasonic bath for 15 min. Bone samples were treated with 20% formic acid for 72 h at 55°C on a shaking machine. The resulting suspension was centrifuged for 5 min at 6,000 g. The resulting suspensions were added to styrene divinyl benzene (SCVB) polymer disposable columns 500 mg/ 6 ml (Bond Elut ENV, Varian). The elution was conducted with a mixture of acidic acetonitrile and diluted formic acid [11]. After addition of internal standard (pradofloxacin-d4), the HPLC analysis was performed as described above.

### Pharmacokinetic and statistical analysis

A non-compartment analysis of serum and ISF data was made. Pharmacokinetic parameters were calculated using the software program WinNonlin 5.2 (Pharsight Corp., USA). Normal distribution of variables (model-residuals) was confirmed by visual assessment of normal probability plots. Comparisons of pharmacokinetic parameters at ISF and serum between doses was calculated by t-test for paired observations. Analyses were carried out with the statistical software SAS, version 9.3 (SAS Institute, Cary, NC).

## Results

**Figure 1:**
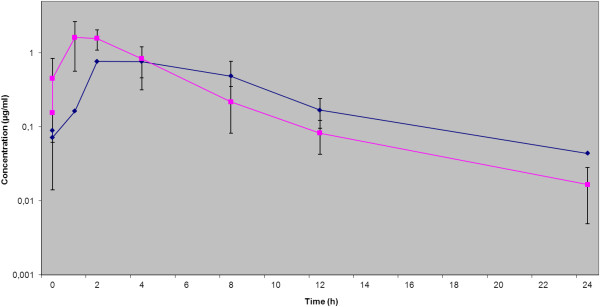
**Serum and ISF concentrations after oral administration of enrofloxacin (5 mg/kg), expressed as mean ± SD.** ISF ♦ Serum ■.

**Figure 2:**
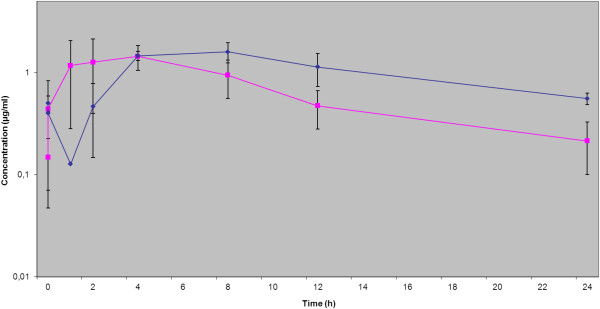
**Serum and ISF concentrations after oral administration of pradofloxacin (3 mg/kg), expressed as mean ± SD.** ISF ♦ Serum ■.

**Figure 3:**
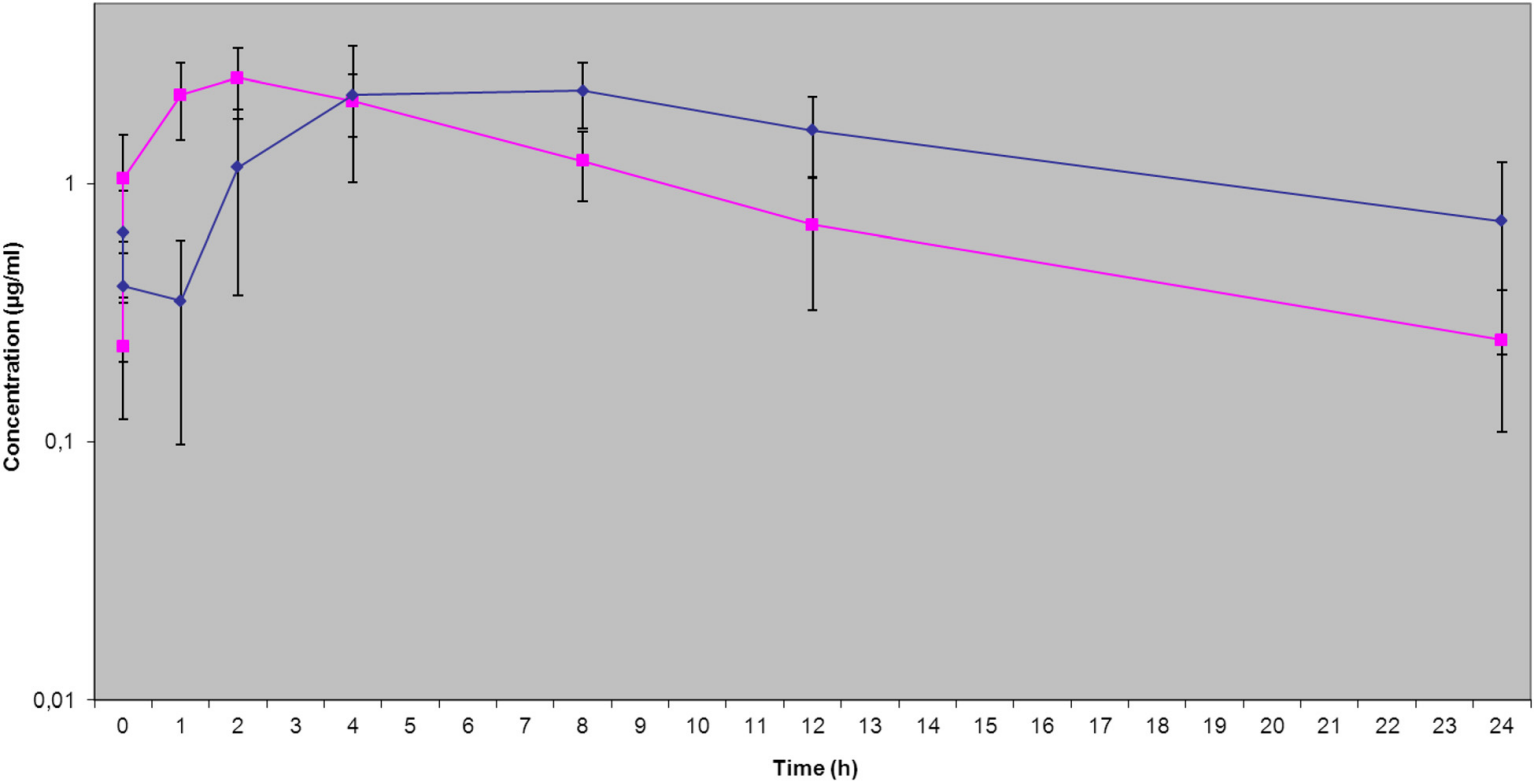
**Serum and ISF concentrations after oral administration of pradofloxacin (6 mg/kg), expressed as mean ± SD.** ISF ♦ Serum ■.

No adverse effects after oral administration of enrofloxacin (5 mg/kg) and pradofloxacin (3 mg/kg and 6 mg/kg, respectively) were observed in any of the dogs. The ultrafiltration procedure was well tolerated in all dogs involved in this study. A total of 288 samples of serum and ISF and 48 double samples of tissue were taken. From these a total of 100% of tissue biopsies (n=48), 89% of serum samples (n=129) and 52 % of ISF samples (n=75) could be evaluated. Among these, for the third treatment (pradofloxacin 6 mg/kg) 95 % of serum samples (n=46), 87 % of ISF samples (n=42) and 100% of tissue biopsies (n=48) were evaluable providing sufficient data for comparative estimation of all three matrices. Serum and ISF concentrations after oral administration of 5 mg/kg enrofloxacin, 3 mg/kg pradofloxacin and 6 mg/kg pradofloxacin were plotted on a semilogarithmic graph for analysis (Figures 1, 2, 3); pharmacokinetic parameters are expressed as the arithmetic mean ± standard deviation in Tables 1, 2, 3.

**Table 1:** Pharmacokinetic parameters (mean ± SD) for enrofloxacin after oral administration (5 mg/kg) in dogs

**Parameter**	**Units**	**PO Serum**	**PO ISF**
**C**_**max**_	μg/ml	1.89±0.83	0.59±0.43
**AUC**_**24**_	h*μg/ml	7.42±3.03	5.19±4.32
**AUC**_**inf**_	h*μg/ml	7.59±3.09	7.54±3.89
**T**_**max**_	h	1.66±0.52	5.33±2.31
**T**_**1/2**_	h	3.18±1.5	4.47±0.42
**T**_**24**_	h	20±6.92	16±6.92
**λ**_**z**_	/h	0.26±0.11	0.16±0.02
**MRT**_**last**_	h	3.70±0.55	7.30±1.14
**MRT**_**inf**_	h	4.04±0.58	8.25±0.79

C_max_ maximum concentration; AUC_24_ area under the curve from time 0 to 24 hours; MRT_last_ mean residence time; λ_z_ first order rate constant of terminal portion of the curve; T_1/2_ half-life; T_max_ time to maximal concentration; T _24_ time of final measurement; AUC_inf_ area under the curve from 0 extrapolated to infinity; MRT_inf_ mean residence time from time 0 extrapolated to infinity for non-infusion models.

**Table 2:** Pharmacokinetic parameters (mean ± SD) for pradofloxacin after oral administration (3 mg/kg) in dogs

**Parameter**	**Units**	**PO Serum**	**PO ISF**
**C**_**max**_	μg/ml	1.85±0.23^*^	1.55±0.31*
**AUC**_**24**_	h*μg/ml	16.18±4.28	21.19±8.12
**AUCi**_**nf**_	h*μg/ml	18.11±4.82^#^	29.95±11.41^#^
**T**_**max**_	h	2.33±1.37^*^	8±3.27*
**T**_**1/2**_	h	6.97±1.83^#^	8.57±2.29^#^
**T**_**24**_	h	24±0	21±6
**λ**_**z**_	/h	0.11±0.03	0.08±0.02
**MRT**_**last**_	h	7.9±0.81	10.35±3.39
**MRT**_**inf**_	h	11.27±3.11	14.78±4.12

C_max_ maximum concentration; AUC_24_ area under the curve from time 0 to 24 hours; MRT_last_ mean residence time; λ_z_ first order rate constant of terminal portion of the curve; T_1/2_ half-life; T_max_ time to maximal concentration; T _24_ time of final measurement; AUC_inf_ area under the curve from 0 extrapolated to infinity; MRT_inf_ mean residence time from time 0 extrapolated to infinity for non-infusion models; * statistical different (p ≤ 0,05); # no statistical difference (p ≤ 0,05).

**Table 3:** Pharmacokinetic parameters (mean ± SD) for pradofloxacin after oral administration (6 mg/kg) in dogs

**Parameter**	**Units**	**PO Serum**	**PO ISF**
**C**_**max**_	μg/ml	2.77±0.63^#^	2.71±0.8^#^
**AUC**_**24**_	h*μg/ml	24.21±5.09	35.19±8.39
**AUCi**_**nf**_	h*μg/ml	26.76±6.27^#^	43.38±17.54^#^
**T**_**max**_	h	2±1.1^*^	6±3.35*
**T**_**1/2**_	h	6.31±1.74^#^	8.35±3.5^#^
**T**_**24**_	h	24±0	24±0
**λ**_**z**_	/h	0.12±0.03	0.09±0.03
**MRT**_**last**_	h	7.16±1.31	10.16±1.83
**MRT**_**inf**_	h	9.58±3.04	14.6±6.41

C_max_ maximum concentration; AUC_24_ area under the curve from time 0 to 24 hours; MRT_last_ mean residence time; λ_z_ first order rate constant of terminal portion of the curve; T_1/2_ half-life; T_max_ time to maximal concentration; T _24_ time of final measurement; AUC_inf_ area under the curve from 0 extrapolated to infinity; MRT_inf_ mean residence time from time 0 extrapolated to infinity for non-infusion models; * statistical different (p ≤ 0,05); # no statistical difference (p ≤ 0,05).

Tissue concentrations after administration of pradofloxacin (6 mg/kg) are given as the arithmetic mean ± standard deviation in Table 4.

**Table 4:** Tissue concentrations (mean± SD) after oral administration (6 mg/kg) of pradofloxacin in dogs (measured at 1-1.5 hours after drug administration)

**Tissue**	**Units**	**Concentration**
**Skin**	μg/mg	0.535±0.290
**Fat**	μg/mg	0.0756±0.0624
**Muscle**	μg/mg	0.709±0.455
**Cartilage**	μg/mg	2.614±1.450
**Bone**	μg/mg	0.361±0.0987
**Liver**	μg/mg	1.501±1.322
**Kidney**	μg/mg	1.435±1.083
**CSF**	μg/ml	0.107±0.063

### ISF values

ISF values of pradofloxacin following oral administration of 3 mg/kg and 6 mg/kg were statistically different for AUC_Inf_ (29.95 ± 11.41 μg* h/ml vs. 43.38 ± 17.54 μg*h/ml, p = 0.0427) and C_max_ (1.55 ± 0.31 μg/ml vs. 2.71 ± 0.8 μg/ml, p = 0.0142). This was not true for T_max_ (8 ± 3.27 h vs. 6 ± 3.35 h, p = 0.1817) and T_1/2_ (8.57 ± 2.29 h vs. 8.35 ± 3.5 h, p = 0.3747). Enrofloxacin oral ISF values reached a mean peak concentration of 0.59 ± 0.43 μg/ml at T_max_ 5.33 ± 2.31 h with T_1/2_ at 4.47 ± 0.42 and an AUC_Inf_ of 7.54 ± 3.89 μg*h/ml.

### Serum values

Oral serum values of pradofloxacin 3 mg/kg and 6 mg /kg were statistically different for AUC_Inf_ (18.11 ± 4.82 μg* h/ml vs. 26.76 ± 6.27 μg*h/ml, p = 0.0040) and C_max_ (1.85 ± 0.23 μg/ml vs. 2.77 ± 0.63 μg/ml, p = 0.0183). T_max_ (2.33 ± 1.37 h vs. 2 ± 1.1 h, p = 0.4650) and T_1/2_ (6.97 ± 1.83 h vs. 6.31 ± 1.74 h (p = 0.4581) showed no statistical difference.

Enrofloxacin oral serum values reached a mean peak concentration of 1.89 ± 0.83 μg/ml at T_max_ 1.66 ± 0.52 h with T_1/2_ at 3.18 ± 1.5 h and an AUC_Inf_ of 7.59 ± 3.09 μg*h/ml.

### Serum and ISF values in comparison

Serum and ISF values of pradofloxacin 3 mg/kg were statistically different for C_max_ (p = 0.0402) and T_max_ (p = 0.0114) but not for AUC_INf_ (p = 0.1682) and T_1/2_ (p = 0.5146). Serum and ISF values of pradofloxacin 6 mg/kg were statistically different for T_max_ (p = 0.0103) but not for C_max_ (p = 0.8071), AUC_INf_ (p = 0.0831) and T_1/2_ (p = 0.2178).

### PK/PD ratios

C_max_/MIC and AUC_24_/MIC ratios were calculated for the label dose of pradofloxacin 3 mg/kg based on MIC_90_-values published by Schink et al (2013) [12] for the bacteria specified in the Veraflox SPC, i.e. *Staphylococcus (pseud)intermedius*, *Escherichia coli* and *Pasteurella multocida* (Table 5).

**Table 5:** Pharmakokinetik/Pharmakodynamic ratios of oral pradofloxacin values (3 mg/kg oral administration) in serum and ISF

	***Pasteurella multocida*(MIC**_**90**_**= 0.015 μg/ml)**		***Escherichia coli*(MIC**_**90**_**= 0.03 μg/ml)**		***Staphylococcus pseudintermedius*(MIC**_**90**_**= 0.12 μg/ml)**	
	**Serum**	**ISF**	**Serum**	**ISF**	**Serum**	**ISF**
C_max_/MIC_90_	123.3	103.3	61.7	51.7	15.4	12.9
AUC_0-24_/MIC_90_	1078.7	1412.7	539.3	706.3	134.8	176.6

### The UF-device

There were some shortcomings affecting the sample collection, which were associated with the UF-device. These included dislocation of the UF-probe followed by the need for re-implantation in four of 18 sampling periods (22%) at the start of the study. Insufficient vacuum resulting in either lack of flow or lack of volume causing drug concentrations below the limit of quantification was assumed for 12 of 18 sampling periods (66%). We assumed that the hub assembly was the critical system component causing these shortcomings. After application of 6 mg/kg pradofloxacin this phenomenon occurred only in two test animals; thus, 42 of 48 (87%) of the samples for this dosing protocol could be utilized.

## Discussion

Bacterial infections mainly occur in the extracellular space. Keeping in mind that from a clinical point of view tissue- and in detail ISF-concentrations are often considered better evidence of drug effectiveness than serum concentrations [13,14] it is the therapeutic aim to apply a dosing scheme that will result in a drug concentration in the ISF above the minimal inhibitory concentration (MIC) [15]. For the newly developed 8-cyano-fluoroquinolone pradofloxacin only few studies to determine drug concentrations in the ISF are available [16,17]. In-vivo UF which was first published in a study by Janle-Swain et al. in 1987 [18] in this regard seems to be a promising alternative to standard methods. In contrast to microdialysis, ultrafiltration establishes an equilibrium across the capillary for the unbound drug concentration as stated theoretically by Ögren & Cars (1985) [19] and being confirmed by Bidgood & Papich (2005) [8] which is essential to directly estimate the relevant concentrations at the site of infection. To the knowledge of the authors, the present study is the first to compare pharmacokinetic data of pradofloxacin based on UF with serum and tissue data from dogs.

Comparison of the serum and ISF concentrations of the reference fluoroquinolone enrofloxacin with the results of Frazier et al. (2000) [20] and those of Bidgood & Papich (2005) [8] showed similar results. This confirmed the validity of the chosen study design and methods. While the kinetics of enrofloxacin displayed a continuous increase of the concentration in the ISF during the absorption phase after application, for pradofloxacin a decrease in concentration was observed initially between the first two measuring time points (more pronounced with the dose 6 mg/kg than with 3 mg/kg) followed by a continuous increase to a maximum drug concentration (Figures 1, 2, 3). It can be hypothesized that this observation is a result of the study design using a multiple dosing regime with lack of a “baseline”. On the other hand a comparatively slightly slower absorption of pradofloxacin may be ruled out, since this effect could not be demonstrated in the analysis of the serum samples. The delay seems to originate from the drug exiting the vascular system into the ISF. However, this only applies to the initial absorption phase; later the increase of the drug concentration became similar for enrofloxacin and pradofloxacin in both doses. What is more due to the shortcomings of the UF-system described later on we abandoned to calculate a lag time for the ISF samples which may also have influenced this phenomenon. The initial concentrations following application of 3 and 6 mg/kg pradofloxacin were always above the MIC_90_ values for most relevant target bacteria which - in context with the proven post-antibiotic effect of pradofloxacin [14,21] confirmed the efficacy of the chosen dosing interval. The maximal drug concentration of pradofloxacin in the ISF (C_max_ μg/ml: 1.55 ± 0.31 for the dose 3 mg/kg exceeded 12.9 to 103.3-fold the MIC_90_ values of 0.015 to 0.12 for the SPC bacterial pathogens *Pasteurella multocida, Escherichia coli* and *Staphylococcus pseudintermedius*[12]. PK/PD integrated models link drug concentrations to their activity on bacterial pathogens [22]. To minimize or prohibit the selection of resistant organisms at present for fluoroquinolones a ratio of the maximum serum concentration to MIC (C_max_/MIC) >10 and a ratio of the area under concentration-time curve over 24 h to MIC (AUC_24_/MIC) >125 [23,24] are widely used. Based on the present study the pradofloxacin standard dosage protocol of 3 mg/kg [25] would exhibit a good efficacy against the label organisms with MIC_90_ of ≤ 0.12 μg/ml. The dosage interval of 24 h thereby was confirmed by the terminal half-life in the serum and ISF for both doses (6.97/6.3 h and 8.5/8.35 h, respectively).

With regard to the protein binding of pradofloxacin of 29 – 37% [26] the suggested adjustment for protein binding in pharmacokinetic-pharmacodynamic assessments [27] seems to be unnecessary in this case due to the fact that calculated unbound drug concentration in serum is lower than the active drug concentration in the ISF. This is similar to results presented by Messenger et al. (2012) [28] for enrofloxacin and may be a phenomenon specific for fluoroquinolones. Although pharmacokinetic analysis of tissue samples is accepted as a standard method to analyse drug distribution in tissue [29], it has several limitations. Since infections addressed by fluoroquinolones are localized in the extracellular space i.e. the ISF, the drug concentrations at site of infection are under- or overestimated when determining them in tissue homogenates [6,30]. The concentrations in tissue samples result from a mixture of different compartments and are thus difficult to interpret [31]. Furthermore, the possibility for continuous and extensive sampling is very limited in the light of ethical considerations. True determination of the maximal tissue concentration is questionable on this basis. The lower tissue concentrations compared to other studies [16] is to be seen in context with the short time span between final drug application and euthanasia of the experimental animals. At the time of euthanasia the concentration development, as shown by the serum and ISF analysis, was generally still in the absorption phase. Over all serum values are a much better predictor of ISF concentrations than total tissular concentrations but ISF-samples proved to illustrate best the relevant concentration values at the site of infection. The advantages of the in-vivo technique of UF have been described in several pharmacokinetic studies. These advantages include data collection directly at the action site of the tested drug in different tissues over a long time period, direct sample analysis without extraction steps, maintenance of an equilibrium during sampling and last but not least reduced strain on the tested animal [19,32,33].

However, the authors recognize some immanent system disadvantages.

The low number of usable samples from the UF (52.08%) could only be assigned to a dislocation or tearing of the membrane-carrying probe in two cases. Most failures were a result of an insufficient vacuum resulting in inadequate ISF flow into the receptive vial. We identified the hub assembly, which consists of three individual components, as the source of the problem. Leakage occurred repeatedly and could not be resolved completely. Because of the unstable negative pressure in the used UF systems during the study duration, we did not arithmetically adjust the resulting time delay during sampling, which would generally be useful for optimal comparability of the serum samples regarding evaluation of the pharmacokinetic parameters [34]. Despite the high number of losses, 87% of the ISF samples could be evaluated for the higher dose rate (pradofloxacin 6 mg/kg).

## Conclusions

In conclusion, peak concentrations detected at the site of infection after oral administration of pradofloxacin using a standard dosage protocol of 3 mg/kg exceed the MIC90 values for indicated and most other bacterial targets. Based on ISF-related PK/PD ratios good clinical efficacy against the bacteria listed in the Veraflox SPC would be predicted. Considering the comparison between serum, ISF and tissue data the authors regard ultrafiltration as the most effective method for pharmacokinetic analysis of fluoroquinolones, since it most realistically reflects the situation at the target site. Furthermore, it is the method with the least invasiveness and stress for the test animal. After elimination of the technical disadvantages of the system, ultrafiltration could become a useful addition to current methods in pharmacokinetics analysis, and perhaps even replace them.

## Competing interests

This study was supported by a grant from Bayer Animal Health GmbH. G. Hauschild received a fee by Bayer Animal Health GmbH for presenting some of the data provided in this manuscript at the 2^nd^ International Veraflox® Symposium in Rome, Nov. 2012. Furthermore the authors declare that they have no competing interests.

## Authors’ contributions

GH conceived of the study and its design, carried out the experimental studies, participated in the statistical analysis and drafted the manuscript. KR participated in the design of the study and carried out the statistical analysis. EE participated in the experimental studies. MS participated in the coordination of the study and in the experimental studies. JH helped to draft the manuscript. GG participated in the design and coordination of the study. All authors read and approved the final manuscript.
